# Relationship between Diet Quality and Periodontal Disease in South Korean Adults Aged ≥40 Years

**DOI:** 10.3390/ijerph20065039

**Published:** 2023-03-13

**Authors:** Mi-Ra Lee

**Affiliations:** Department of Dental Hygiene, Hanseo University, Seosan 31962, Republic of Korea; leemra1@hanmail.net; Tel.: +82-41-660-1576

**Keywords:** diet quality, national health and nutrition survey, nutrition, oral health, periodontal disease

## Abstract

The purpose of this study was to investigate the relationship between diet quality and periodontal disease, in adults aged ≥40 years, using data from the 7th (2016–2018) Korea National Health and Nutrition Survey (KNHANES), representing South Koreans. The subjects of this study were 7935 people aged ≥40 years, who responded to the items in the Korea Healthy Eating Index (KHEI) and underwent periodontal examination. Complex sample univariate and multivariate logistic regression analyses were conducted, to analyze the relationship between the diet quality and periodontal disease. The group with a low diet quality for energy intake balance, showed a higher risk of periodontal disease than the group with a high diet quality for energy intake balance, and it was confirmed that the diet quality in adults aged ≥40 years was related to periodontal disease. Therefore, regular diet evaluations, and the counseling of gingivitis and periodontitis patients by dental experts, will have a positive effect on the restoration and improvement of periodontal health in adults.

## 1. Introduction

Periodontal disease is a disease which affects the supporting tissues of teeth. When the periodontal pocket deepens, as inflammation occurs in the periodontal tissue, the loss of alveolar bone, tooth mobility, and periodontal abscesses can occur. If periodontal disease is left untreated, it will eventually lead to tooth loss and a lower quality of life [1]. In previous studies, periodontal disease has been reported to be caused by classic periodontal pathogens such as *Porphyromonas gingivalis*, environmental factors, complex interactions of the host’s immune system, the host’s oral health behaviors, and systemic diseases, and the imbalance between them causes periodontitis to develop and progress [2,3,4,5]. To treat periodontal disease, the mechanical removal of biofilm, to improve oral hygiene, antibiotics, and surgical treatment can be applied [6,7]. Furthermore, as a method to prevent and manage periodontal disease, healthy lifestyle changes can be made, such as smoking cessation, stress reduction, engagement in physical activity, brushing teeth, and regular dental checkups [2]. In addition to these factors, adherence to a healthy and balanced diet plays an important role in maintaining and promoting periodontal health [8], and it also lowers the risk of many inflammatory diseases such as cardiovascular disease, type 2 diabetes, etc. [5].

Macronutrients such as various carbohydrates, proteins, and fats contained in food, and micronutrients such as vitamins and minerals, affect a host’s oral health and periodontal condition by acting on their inflammatory and immune responses. In other words, a balanced intake of unprocessed complex carbohydrates, vegetable proteins, omega-3 fatty acids, vitamins, and minerals has a positive effect on periodontal health, as oxidative stress is removed through redox-cycling reactions by antioxidant nutrients, while the excessive intake of refined carbohydrates, non-vegetable proteins, and saturated fatty acids may increase oral inflammation, by stimulating inflammation through the production of ROS (H_2_O_2_, HOCL, OH^+^) and oxidative stress [9]. Previous studies examining the relationship between dietary intake and periodontal health showed that those with a vegan diet had lower rates of gingival inflammation and better oral hygiene [10], and that, regardless of traditional risk factors, gingival and periodontal inflammation was associated with high-glycemic food consumption alone [11]. It has also been reported that whole grain consumption, without increasing total energy intake, may reduce the risk of gingivitis and periodontitis [12]. Furthermore, it has been demonstrated that periodontal inflammation and periodontal pocket depth are reduced with the intake of omega-3 fatty acids and supplements [13].

As such, studies regarding diet or nutrition related to periodontal disease have recently been reported [10,11,12,13]. However, studies targeting the middle-aged and older age groups, in which the prevalence of chronic diseases is increasing, are lacking. The prevalence of periodontal disease also tends to increase in adults after the age of 40, and high-quality dietary intake would be very important in managing their chronic diseases, including periodontal disease. Therefore, the purpose of this study was to evaluate whether or not there is a relationship between the quality of diet and periodontal disease in adults aged ≥40 years, using the data from the 7th (2016–2018) Korea National Health and Nutrition Survey (KNHANES), which represents South Koreans.

## 2. Materials and Methods

### 2.1. Participants

**Figure 1:**
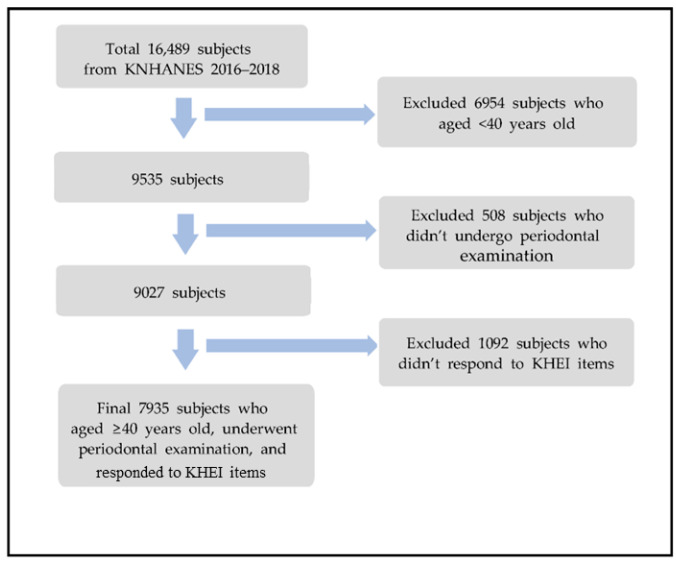
Flow chart for selecting process of study subject.

This study was conducted using the data from the 7th (2016–2018) KNHANES of the Korea Centers for Disease Control and Prevention. The KNHANES uses the most recent population and housing survey data available at the time of sample design as a basic sampling frame, the sampling method is a two-step stratified colony sampling method, and survey districts and households are extracted first and second. In the case of the 7th (2016–2018) KNHANES, the extraction frame was stratified based on city/province, dong/eup/myeon, and housing type (general house, or apartment), and the ratio of residential area and the ratio of household heads’ educational attainment, were used as the implicit stratification criteria. Within the sample households, all household members aged ≥1 year, who met the appropriate household member requirements, were selected as survey subjects [14]. A total of 16,489 subjects participated in the 7th (2016–2018) KNHANES. 6954 subjects who aged <40 years old were excluded. Participants who didn’t undergo periodontal examination (*n* = 508) nor responded to the Korea Healthy Eating Index (KHEI) items (*n* = 1092) were excluded. The final subjects extracted for this study were 7935 individuals (Figure 1). The study was conducted according to the guidelines of the Declaration of Helsinki and approved by the Institutional Review Board of Hanseo University (IRB NO. HS23-01-01).

### 2.2. Measurements

The KNHANES collects survey data through household member confirmation surveys, health surveys, medical examinations, and nutrition surveys. As independent variables in this study, socio-demographic characteristics (sex, age, household income, education level, and marital status) and oral health behavior variables (tooth brushing frequency per day, use of oral hygiene devices, dental examination within one year, and smoking and drinking), from the health questionnaire of the KNHANES, were used, and general health status variables (the prevalence of hypertension, diabetes, and obesity) were used from the medical examinations. In addition, the KHEI variables calculated from the food intake frequency survey and the 24 h recall survey of the nutrition survey were used. The KHEI [15], developed for Korean adults, is an index that can evaluate diet quality, and it is divided into three areas and consists of a total of 14 sub-items. The first area is the ‘recommended foods and appropriateness evaluation area’, which consists of eight sub-items (whether you have breakfast, mixed grain intake, total fruit intake, fresh fruit intake, total vegetable intake, intake of vegetables excluding kimchi and pickles, intake of meat, fish, eggs, and legumes, and intake of milk and dairy products). When breakfast was consumed 5–7 times a week, the consumption of meat, fish, eggs, and legumes was ≥5 units per day for males, or ≥4 units per day for females, and the consumption of milk and dairy products was ≥1 unit per day, the highest score was evaluated as 10 points. When the mixed grain intake was ≥0.3 times a day, the total fruit intake was ≥3 units per day for males, or ≥2 units per day for females, the fresh fruit intake was ≥1.5 units per day for males, or ≥1 unit per day for females, the total vegetable intake was ≥8 units per day, and the intake of vegetables excluding kimchi and pickles was ≥5 units per day, the highest score was 5. The second area is the ‘moderation evaluation area’, which consists of three sub-items (rate of energy intake from saturated fatty acids, sodium intake, and sweets and beverages energy intake rate). Among the saturated fatty acid ratios, when fatty acids made up ≤7% of the total energy, this was given the highest score of 10, and >10% equated to the lowest score of 0; and for sodium intake, the highest score was 10 when the intake was ≤2000 mg per day, and the lowest score was 0 when the intake was >6500 mg. For sweets and beverages, the highest score was 10 when sweets and beverages made up ≤10% of the total energy intake, and the lowest was 0 when sweets and beverages made up >20%. The third area is the ‘energy intake balance evaluation area’, which consists of three sub-items (carbohydrate energy intake rate, fat energy intake rate, and energy appropriate intake). When the carbohydrate energy intake rate was 55–65%, the fat energy intake rate was 10–30%, and the energy intake was 75–125% of the estimated required amount, the highest score was 5 points [16]. The KHEI has a total score of 100, and higher scores mean a higher quality of food intake. Even if the total score is the same, the degree of the effect on health is different according to the score distribution of sub-items [17]. As a dependent variable, periodontal tissue examination variables were used from the oral examination of the KNHANES. Periodontal examinations to evaluate the prevalence of periodontal tissue were conducted based on the Community Periodontal Index (CPI) of the World Health Organization (WHO). The number of teeth to be examined was 10, numbered 11, 16, 17, 26, 27, 31, 36, 37, 46, and 47, and the test result was recorded as 6 teeth. The examination criteria for periodontal tissue were classified into 0 points for healthy periodontal tissue, 1 point for bleeding periodontal tissue, 2 points for calculus-forming periodontal tissue, 3 points for periodontal tissue with 4–5 mm periodontal pockets, and 4 points for periodontal tissue with periodontal pockets of ≥6 mm. According to the periodontal disease classification criteria, if periodontal tissues were recorded as ≥3 points, they were classified as having periodontal disease [14].

### 2.3. Statistical Analyses

Since the KNHANES was sampled using the two-step stratified cluster sampling method, the data analysis in this study reflected the contents of the complex sampling design considering the complex sample elements of strata, colonies, and weights. To analyze the relationship between subject characteristics and periodontal disease by sex, a complex samples chi-square test was conducted. Additionally, a complex samples *t*-test was used to analyze the relationship between periodontal disease and the KHEI by sex. To assess KHEI related to periodontal disease, a complex sample logistic regression analysis was performed. Two models were run in logistic regression analyses. In Model I, univariate logistic regression analysis was performed. In Model II, multivariate logistic regression analysis was performed after adjusting for sex, age, household income, education level, marital status, tooth brushing frequency per day, the use of oral hygiene devices, dental examination within one year, smoking, hypertension, diabetes, and obesity, which were statistically significant variables in the complex samples chi-square test (*p* < 0.05). In this analysis, based on the median value, the total score of KHEI was classified as <67 or ≥67, and in the three areas, ‘recommended food and adequacy evaluation area’ was classified as <34 or ≥34, ‘moderation evaluation area’ was classified as <26 or ≥26, and the ‘energy intake balance evaluation area’ was classified into two groups, of <9 and ≥9. For data analysis, the IBM SPSS 20.0 program (SPSS Inc., Chicago, IL, USA) was used, and statistical significance was determined at a significance level of 0.05.

## 3. Results

### 3.1. Relationship between Subject Characteristics and Periodontal Disease by Sex

As a result of analyzing the relationship between subject characteristics and periodontal disease by sex, it was found that in the ‘60s’ age group, ‘lower to middle’ household income, ‘less than high school graduate’ education level, and ‘widowed or divorced’ marital status, brushing <3 times a day, not using oral hygiene devices, not having dental examination within one year, current smoking, and having hypertension and diabetes groups, both males and females showed a higher prevalence of periodontal disease than in the other groups. In addition, only females showed a higher prevalence of periodontal disease in the obese group than in the normal or low- weight groups (*p* < 0.05) (Table 1).

### 3.2. Relationship between Periodontal Disease and KHEI by Sex

As a result of analyzing the relationship between periodontal disease and the KHEI by sex, in males, the group with periodontal disease, compared to the group without periodontal disease, had lower average scores in the KHEI’s recommended food and adequacy evaluation area, total fruit intake, vegetable intake except for kimchi and pickles, meat, fish, egg, and legume intake. In addition, the group with periodontal disease had a higher average score in the moderation evaluation area and saturated fatty acid energy intake ratio, and a lower average score in the fat energy intake ratio, than the group without periodontal disease. In females, the group with periodontal disease, compared to the group without periodontal disease, had lower average scores in the total score of the KHEI, the recommended food and adequacy evaluation area, meat, fish, egg, and legume intake, and milk and dairy product intake. In addition, the group with periodontal disease had a higher average score in the moderation evaluation area and saturated fatty acid energy intake ratio, and a lower average score in the energy intake balance area, and the carbohydrate and fat energy intake ratio, than the group without periodontal disease (*p* < 0.05) (Table 2).

### 3.3. KHEI Related to Periodontal Disease

As a result of analyzing the KHEI related to periodontal disease, in Model I, the group with a total KHEI score of <67 was at a 1.21 times higher risk of periodontal disease than the group with a total score of ≥67, and the group with a score of <34 in the recommended food and adequacy evaluation area was at a 1.20 times higher risk of periodontal disease than the group with a score of ≥34. It was found that the group with a score of <9 in the energy intake balance areas had a 1.39 times higher risk of periodontal disease than the group with a score of ≥9. In Model Ⅱ, after adjusting for sex, age, household income, education level, marital status, tooth brushing frequency per day, the use of oral hygiene devices, dental examination within one year, smoking, hypertension, diabetes, and obesity, the group with a score of <9 in the energy intake balance areas showed a 1.15 times higher risk of periodontal disease than the group with a score of ≥9 (*p* < 0.05) (Table 3).

## 4. Discussion

Periodontal disease is the sixth most prevalent chronic disease in the world and the first disease to cause tooth loss in adults [18]. Although nutrition is not considered a risk factor for periodontal disease, it is an essential component for the prevention of periodontal disease, including several chronic diseases [19], and it has a significant impact on the maintenance of the body’s immune response in an optimal functioning state [20].

In this study, when the relationship between periodontal disease and KHEI was analyzed, both males and females with periodontal disease showed lower average scores for protein intake, such as meat, fish, eggs, and legumes, among the KHEI’s recommended food and adequacy evaluation area, than the group without periodontal disease. This was similar to the results of a study by Hwang and Park [21], in which it was shown that the amount and quality of protein intake in Korean adults had an effect on periodontal disease. Although the role of proteins in systemic inflammation is not yet well defined, malnutrition, particularly protein malnutrition, accompanied by deficiencies in antioxidant nutrients, leads to impaired cytokine production and cellular function, a reduced acute-phase protein response that plays a central role in promoting healing, endocrinopathy, an impaired drug metabolism, and an impaired response to stress [22]. Therefore, the reduction in oxidative stress due to the increased intake of antioxidants through one’s diet, will have a positive effect on relieving gingival and periodontal inflammation, as the host’s immune response is regulated [23].

In males, compared to the group without periodontal disease, the group with periodontal disease showed lower average scores in terms of the total fruit intake and vegetable intake except for kimchi and pickles, in the KHEI’s recommended food and adequacy evaluation area. Salazar et al. [24] reported that there was an inverse relationship between fruit intake and periodontal disease, whereas Nielsen et al. [25] reported that periodontal disease in older adults was not associated with low fruit and vegetable intake. Similarly, in the results of this study, fruit and vegetable intake was found to be related to periodontal disease in males, but no relationship was found in females. Vegetarians may have better periodontal health with less oral inflammatory symptoms and periodontal damage, given that they avoid foods such as red meat and processed foods, and pursue an overall healthier lifestyle [26]. Additionally, fruit and vegetables are excellent sources of antioxidants, which may have a positive and protective effect on periodontal health [9].

In addition, in the results of this study, the total score of the KHEI was lower in the group with periodontal disease than in the group without periodontal disease in females, confirming the low quality of food intake. This is consistent with the report by Salazar, C.R. et al. [24], which showed that the higher the quality of the diet, the lower the probability of severe periodontitis, and the results of an 11-year follow-up study by Jauhiainen, L.M. et al. [27], which showed that a poor quality diet was associated with the development of periodontal disease in middle-aged adults. A healthy and balanced diet or nutritional intake have anti-inflammatory and protective effects on periodontal health [9], and play an important role in maintaining the symbiosis between oral microflora and periodontal health [28]. Diet can have direct and indirect effects on periodontitis, through very complex biological mechanisms. Furthermore, diet quality is associated with inflammation or diseases such as metabolic syndrome [29], atopic dermatitis [16], type 2 diabetes, cardiovascular disease, and rheumatoid arthritis [5], suggesting that it affects not only oral disease but also systemic health. Therefore, patients with oral and systemic diseases should be especially encouraged to have proper dietary control and nutritional intake. In addition, in the results of this study, oral health behaviors such as tooth brushing frequency per day, the use of oral hygiene devices, dental examinations within one year, and smoking were found to be variables related to periodontal disease, so a lifestyle for correct health and oral health is essential, along with proper dietary control.

For males, the group with periodontal disease had a lower average score of fat energy intake ratio in the energy intake balance evaluation area of the KHEI, and for females, the group with periodontal disease had a lower average score for carbohydrate and fat energy intake ratios than the group without periodontal disease. In addition, as a result of a logistic regression analysis on the KHEI related to periodontal disease, in Model Ⅱ, the group with a score of <9 in the energy intake balance area had a 1.15 times higher risk of periodontal disease than the group with a score of ≥9, after adjusting for sex, age, household income, education level, marital status, tooth brushing frequency per day, the use of oral hygiene devices, dental examination within one year, smoking, hypertension, diabetes, and obesity. This finding was similar to studies by Woelber, J.P. et al. and Martinon, P. et al., in which it was reported that the risk of gingival infection was reduced by about half when carbohydrate intake was restricted in the diet for four weeks [30], and that high sugar, high saturated fat, low polyol, low fiber, and low polyunsaturated fat intake increased the risk of periodontal disease, as found by [19]. Carbohydrates and fats are essential macronutrients, that are used as components and energy sources for the human body. However, the excessive intake of sugar or refined carbohydrates induces an inflammatory response and promotes the growth of microorganisms that cause periodontal disease [19], which can change microbial diversity and directly affect periodontal ligament cells [31]. Moreover, high-glycemic-index carbohydrates appear to directly promote inflammatory processes, through the activation of the redox-sensitive transcription factor NF-κB and oxidative stress, and this mechanism may explain the link between carbohydrates, the glycemic index, and chronic disease [32]. It is also speculated that a saturated-fat-rich diet increases the intensity and duration of inflammatory processes as well as oxidative stress [33], promoting periodontal damage [31]. However, omega-3 fatty acids (polyunsaturated fatty acids) have a positive effect on periodontal disease [19,33]. Therefore, a diet low in carbohydrates, rich in omega-3 fatty acids, and rich in vitamins C and D and fiber, may have a positive effect on relieving gingival and periodontal inflammation [30]. Commonly known healthy diets include the Mediterranean, DASH, vegetarian, and Okinawan diets. A diet optimized for oral health can significantly reduce gingival and periodontal inflammation to a clinically important extent, and furthermore, it may reduce the risk of diabetes, cancer, and cardiovascular disease [19,30].

The limitations of this study are that it is difficult to determine the temporal causal relationship between diet quality and periodontal disease, because it was designed as a cross-sectional study. In addition, since data from the 24 h recall survey of the KNHANES were used to evaluate the diet quality, it is difficult to determine the subject’s general dietary intake pattern. Additionally, since the data were self-reported, there may be measurement errors. Furthermore, in diagnosing periodontal disease, only the CPI criteria were evaluated, without reflecting the criteria of probing pocket depth and clinical attachment loss. The periodontal pocket depth used in the CPI does not necessarily reflect the amount of periodontal tissue loss, and since only designated teeth are examined, not all teeth, there is a possibility of underestimating or overestimating periodontal disease. Despite these limitations, this study is significant, in that it identified the relationship between the diet quality and periodontal disease in adults aged ≥40 years using the data from the KNHANES, which is representative of the Korean population. Accordingly, dentists and dental hygienists should regularly evaluate adult patients’ diet and nutritional status and provide nutritional counseling, to maintain the optimal functioning of the body’s immune system and promote optimal periodontal health.

## 5. Conclusions

This study analyzed the relationship between diet quality and periodontal disease in adults aged ≥40 years, using data from the 7th (2016–2018) KNHANES, representing South Koreans. The group with a low diet quality for energy intake balance showed a higher risk of periodontal disease than the group with a high diet quality for energy intake balance, and it was found that the diet quality in adults aged ≥40 years was related to periodontal disease. In conclusion, it is necessary for dental experts to regularly check patients’ diets and provide counseling for appropriate dietary control, in order to improve adult patients’ periodontal health.

## Figures and Tables

**Table 1:** Relationship between subject characteristics and periodontal disease by sex.

Characteristics	Division	Male			Female		
		With Periodontal Disease	Without Periodontal Disease	*p* *	With Periodontal Disease	Without Periodontal Disease	*p* *
Socio-demographic characteristics							
Age (years)	40–49	296 (37.3)	509 (62.7)	<0.001	228 (15.8)	1050 (84.2)	<0.001
	50–59	448 (50.1)	414 (49.9)		411 (32.6)	845 (67.4)	
	60–69	463 (55.5)	374 (44.5)		443 (39.5)	648 (60.5)	
	≥70	409 (53.5)	341 (46.5)		475 (45.2)	581 (54.8)	
Household income	1st and 2nd quartile	804 (54.7)	669 (45.3)	<0.001	927 (38.8)	1403 (61.2)	<0.001
	3rd and 4th quartile	807 (44.9)	966 (55.1)		627 (25.7)	1717 (74.3)	
Education level	≤High school	1068 (53.9)	915 (46.1)	<0.001	1274 (36.3)	2119 (63.7)	<0.001
	≥College	451 (39.9)	658 (60.1)		226 (19.5)	884 (80.5)	
Marital status	Married	1363 (48.6)	1413 (51.4)	0.013	1020 (28.3)	2367 (71.7)	<0.001
	Single	79 (44.1)	99 (55.9)		26 (25.8)	77 (74.2)	
	Widowed or divorced	174 (58.1)	216 (41.9)		511 (43.2)	678 (56.8)	
Oral health behaviors							
Tooth brushing frequency	<3 times	977 (52.8)	865 (47.2)	<0.001	819 (37.0)	1349 (63.0)	<0.001
	≥3 times	625 (44.8)	762 (55.2)		722 (27.8)	1745 (72.2)	
Use of oral hygiene devices	Yes	670 (45.1)	800 (54.9)	0.001	677 (25.4)	1876 (74.6)	<0.001
	No	932 (52.8)	827 (47.2)		865 (40.5)	1219 (59.5)	
Dental examination within 1 year	Yes	538 (44.5)	657 (55.5)	<0.001	439 (24.1)	1254 (75.9)	<0.001
	No	1064 (52.1)	970 (47.9)		1103 (36.6)	1841 (63.4)	
Smoking	Yes	576 (57.0)	414 (43.0)	<0.001	82 (43.4)	90 (56.6)	0.011
	No	1026 (45.9)	1213 (54.1)		1460 (31.5)	3006 (68.5)	
Drinking	Yes	1094 (49.4)	1099 (50.6)	0.818	499 (29.9)	1086 (70.1)	0.058
	No	510 (48.9)	529 (51.1)		1046 (33.1)	2009 (66.9)	
Health status							
Hypertension	Normal	413 (46.2)	475 (53.8)	0.001	450 (23.7)	1365 (76.3)	<0.001
	Prehypertension	417 (45.0)	492 (55.0)		363 (32.8)	716 (67.2)	
	Hypertension	782 (53.5)	669 (46.5)		741 (40.6)	1041 (59.4)	
Diabetes	Normal	657 (46.1)	769 (53.9)	<0.001	784 (28.3)	1973 (71.7)	<0.001
	Impaired fasting	516 (49.2)	523 (50.8)		391 (34.2)	652 (65.8)	
	Diabetes	356 (58.0)	256 (42.0)		283 (44.8)	329 (55.2)	
Obesity	Normal	914 (48.8)	946 (51.2)	0.881	880 (29.4)	2047 (70.6)	<0.001
	Low weight	39 (50.9)	35 (49.1)		39 (24.3)	96 (75.7)	
	Obesity	659 (49.7)	653 (50.3)		631 (37.3)	973 (62.7)	

* By complex samples chi-square test.

**Table 2:** Relationship between periodontal disease and KHEI by sex.

KHEI	Male			Female		
	With Periodontal Disease	Without Periodontal Disease	*p* *	With Periodontal Disease	Without Periodontal Disease	*p* *
Total	64.16 ± 0.38	65.02 ± 0.41	0.105	66.28 ± 0.44	67.69 ± 0.29	0.004
Adequacy						
Breakfast	8.52 ± 0.10	8.43 ± 0.09	0.491	8.46 ± 0.10	8.26 ± 0.08	0.125
Mixed grains	2.23 ± 0.07	2.35 ± 0.07	0.196	2.32 ± 0.07	2.25 ± 0.05	0.379
Total fruit	2.05 ± 0.07	2.27 ± 0.07	0.021	2.86 ± 0.08	2.98 ± 0.06	0.155
Fresh fruit	2.28 ± 0.07	2.44 ± 0.07	0.105	3.09 ± 0.08	3.15 ± 0.06	0.491
Total vegetables	3.90 ± 0.04	3.95 ± 0.04	0.332	3.49 ± 0.05	3.45 ± 0.03	0.454
Vegetables, excluding kimchi and pickles	3.32 ± 0.05	3.53 ± 0.05	0.002	3.20 ± 0.05	3.27 ± 0.04	0.233
Meat, fish, eggs, and legumes	6.90 ± 0.09	7.29 ± 0.10	0.002	6.47 ± 0.11	6.79 ± 0.07	0.012
Milk and dairy	2.45 ± 0.13	2.71 ± 0.12	0.136	2.92 ± 0.14	3.52 ± 0.11	<0.001
Subtotal	31.64 ± 0.32	32.98 ± 0.33	0.003	32.82 ± 0.36	33.68 ± 0.25	0.034
Moderation						
Saturated fatty acid	8.49 ± 0.09	8.07 ± 0.11	0.001	8.46 ± 0.10	8.00 ± 0.08	<0.001
Sodium	6.12 ± 0.10	5.82 ± 0.12	0.054	7.99 ± 0.09	7.88 ± 0.06	0.305
Sweets and beverages	9.42 ± 0.05	9.40 ± 0.07	0.820	9.33 ± 0.07	9.35 ± 0.05	0.756
Subtotal	24.02 ± 0.15	23.30 ± 0.18	0.001	25.78 ± 0.14	25.23 ± 0.16	0.003
Balance						
Carbohydrate	2.25 ± 0.06	2.42 ± 0.07	0.052	1.92 ± 0.07	2.40 ± 0.05	<0.001
Fat	3.07 ± 0.06	3.26 ± 0.07	0.032	2.72 ± 0.07	3.25 ± 0.05	<0.001
Energy intake	3.18 ± 0.07	3.07 ± 0.07	0.271	3.04 ± 007	3.14 ± 0.04	0.229
Subtotal	8.50 ± 0.13	8.75 ± 0.15	0.201	7.68 ± 0.15	8.79 ± 0.10	<0.001

* By complex samples *t*-test.

**Table 3:** KHEI related to periodontal disease.

KHEI	Division	Model Ⅰ		Model Ⅱ	
		ORs (CI)	*p* *	AORs (CI)	*p* *
Total	<67	1.21 (1.08–1.36)	0.001	1.06 (0.93–1.21)	0.386
	≥67 (ref.)	1.00		1.00	
Adequacy	<34	1.20 (1.07–1.36)	0.002	1.05 (0.92–1.21)	0.451
	≥34 (ref.)	1.00		1.00	
Moderation	<26	0.94 (0.84–1.05)	0.302	1.01 (0.89–1.15)	0.831
	≥26 (ref.)	1.00		1.00	
Balance	<9	1.39 (1.25–1.54)	<0.001	1.15 (1.02–1.29)	0.019
	≥9 (ref.)	1.00		1.00	

OR: odds ratio (95% confidence interval). * by complex samples logistics regression. Model Ⅰ: unadjusted model. Model Ⅱ: adjusted by sex, age, household income level, education level, marital status, tooth brushing frequency per day, use of oral hygiene devices, dental examination within one year, smoking, hypertension, diabetes, and obesity.

## Data Availability

The National Health and Nutrition Survey data disclose raw data on the Korea Centers for Disease Control and Prevention’s website, so consent to the collection and use of personal information and data was downloaded and used after user information registration (URL: https://knhanes.kdca.go.kr/knhanes/sub03/sub03_02_05.do, accessed on 1 August 2022).

## References

[1] Lee J.H., Hwang T.Y. (2018). Effects of multiple chronic diseases on periodontal disease in Korean adults. J. Agric. Med. Community Health.

[2] Li A., Chen Y., Schuller A.A., van der Sluis L.W.M., Tjakkes G.E. (2021). Dietary inflammatory potential is associated with poor periodontal health: A population-based study. J. Clin. Periodontol..

[3] Peres M.A., Macpherson L.M.D., Weyant R.J., Daly B., Venturelli R., Mathur M.R., Listl S., Celeste R.K., Guarnizo-Herreño C.C., Cristin Kearns C. (2019). Oral diseases: A global public health challenge. Lancet.

[4] Zhang S., Yu N., Arce R.M. (2020). Periodontal inflammation: Integrating genes and dysbiosis. Periodontology 2000.

[5] Van der Velden U., Kuzmanova D., Chapple I.L. (2011). Micronutritional approaches to periodontal therapy. J. Clin. Periodontol..

[6] Keestra J.A., Grosjean I., Coucke W., Quirynen M., Teughels W. (2015). Non-surgical periodontal therapy with systemic antibiotics in patients with untreated chronic periodontitis: A systematic review and meta-analysis. J. Periodontal Res..

[7] Sanz M., Herrera D. (2020). Treatment of stage I-III periodontitis-The EFP S3 level clinical practice guideline. J. Clin. Periodontol..

[8] Najeeb S., Zafar M.S., Khurshid Z., Zohaib S., Almas K. (2016). The role of nutrition in periodontal health: An update. Nutrients.

[9] Santonocito S., Polizzi A., Palazzo G., Indelicato F., Isola G. (2021). Dietary factors affecting the prevalence and impact of periodontal disease. Clin. Cosmet. Investig. Dent..

[10] Laffranchi L., Zotti F., Bonetti S., Dalessandri D., Fontana P. (2010). Oral implications of the vegan diet: Observational study. Minerva Stomatol..

[11] Lula E.C., Ribeiro C.C., Hugo F.N., Alves C.M., Silva A.A. (2014). Added sugars and periodontal disease in young adults: An analysis of NHANES III data. Am. J. Clin. Nutr..

[12] Merchant A.T., Pitiphat W., Franz M., Joshipura K.J. (2006). Whole-grain and fiber intakes and periodontitis risk in men. Am. J. Clin. Nutr..

[13] Doğan B., Kemer Doğan E.S., Özmen Ö., Fentoğlu Ö., Kırzıoğlu F.Y., Calapoğlu M. (2022). Synergistic effect of omega-3 and probiotic supplementation on preventing ligature-induced periodontitis. Probiotics Antimicrob. Proteins.

[14] The Seventh (2016–2018) Korea National Health and Nutrition Examination Survey Analysis Guideline. https://knhanes.cdc.go.kr/knhanes/sub03/sub03_06_02.do.

[15] Yook S.M., Park S., Moon H.K., Kim K., Shim J.E., Hwang J.Y. (2015). Development of Korean healthy eating index for adults using the Korea National Health and Nutrition Examination Survey data. J. Nutr. Health.

[16] Kim H.W., Kim J.M. (2022). Relationship between atopic dermatitis and the Korean Healthy Eating Index score of adults: Based on the 7th (2016–2018) Korea National Health and Nutrition Examination Survey. J. Nutr. Health.

[17] Reedy J., Lerman J.L., Krebs-Smith S.M., Kirkpatrick S.I., Pannucci T.E., Wilson M.M., Subar A.F., Kahle L.L., Tooze J.A. (2018). Evaluation of the Healthy Eating Index-2015. J. Acad. Nutr. Diet..

[18] Balta M.G., Papathanasiou E., Blix I.J., Van Dyke T.E. (2021). Host modulation and treatment of periodontal disease. J. Dent. Res..

[19] Martinon P., Fraticelli L., Giboreau A., Dussart C., Bourgeois D., Carrouel F. (2021). Nutrition as a key modifiable factor for periodontitis and main chronic diseases. J. Clin. Med..

[20] Boyd L.D., Madden T.E. (2003). Nutrition, infection, and periodontal disease. Dent. Clin. N. Am..

[21] Hwang S.Y., Park J.E. (2022). The effects of dietary protein intake and quality on periodontal disease in Korean adults. J. Korean Soc. Dent. Hyg..

[22] Enwonwu C.O. (1995). Interface of malnutrition and periodontal diseases. Am. Clin. Nutr..

[23] Chapple I.L. (2009). Potential mechanisms underpinning the nutritional modulation of periodontal inflammation. J. Am. Dent. Assoc..

[24] Salazar C.R., Laniado N., Mossavar-Rahmani Y., Borrell L.N., Oi Q., Sotres-Alvarez D., Morse D.E., Singer R.H., Kaplan R.C., Badner V. (2018). Better-quality diet is associated with lower odds of severe periodontitis in US Hispanics/Latinos. J. Clin. Periodontol..

[25] Nielsen S.J., Trak-Fellermeier M.A., Joshipura K., Dye B.A. (2016). Dietary fiber intake is inversely associated with periodontal disease among US adults. J. Nutr..

[26] Staufenbiel I., Weinspach K., Förster G., Geurtsen W., Günay H. (2013). Periodontal conditions in vegetarians: A clinical study. Eur. J. Clin. Nutr..

[27] Jauhiainen L.M., Ylöstalo P.V., Knuuttila M., Männistö S., Kanerva N., Suominen A.L. (2020). Poor diet predicts periodontal disease development in 11-year follow-up study. Community Dent. Oral Epidemiol..

[28] Kato I., Vasquez A., Moyerbrailean G., Land S., Djuric Z., Sun J., Lin H.S., Ram J.L. (2017). Nutritional correlates of human oral microbiome. J. Am. Coll. Nutr..

[29] Choi S.A., Chung S.S., Rho J.O. (2022). Benefits of adherence to the Korea Healthy Eating Index on the risk factors and incidence of the metabolic syndrome: Analysis of the 7th (2016–2018) Korea National Health and Nutrition Examination Survey. J. Nutr. Health.

[30] Woelber J.P., Bremer K., Vach K., König D., Hellwig E., Ratka-Krüger P., Al-Ahmad A., Tennert C. (2016). An oral health optimized diet can reduce gingival and periodontal inflammation in humans—A randomized controlled pilot study. BMC Oral Health.

[31] Altun E., Walther C., Borof K., Petersen E., Lieske B., Kasapoudis D., Navid Jalilvand N., Beikler T., Jagemann B., Zyriax B.-C. (2021). Association between dietary pattern and periodontitis—A cross-sectional study. Nutrients.

[32] Dickinson S., Hancock D.P., Petocz P., Ceriello A., Brand-Miller J. (2008). High-glycemic index carbohydrate increases nuclear factor-kappaB activation in mononuclear cells of young, lean healthy subjects. Am. J. Clin. Nutr..

[33] Varela-López A., Giampieri F., Bullón P., Battino M., Quiles J.L. (2016). Role of lipids in the onset, progression and treatment of periodontal disease. A systematic review of studies in humans. Int. J. Mol. Sci..

